# A survey of prescribing practices by general dentists in Australia

**DOI:** 10.1186/s12903-019-0882-6

**Published:** 2019-08-22

**Authors:** L. Teoh, R. J. Marino, K. Stewart, M. J. McCullough

**Affiliations:** 1 Melbourne Dental School, The University of Melbourne, 720 Swanston Street, Carlton, VIC 3010 Australia; 2 Centre for Medicine Use and Safety, Monash University, Parkville, VIC Australia

**Keywords:** Antimicrobial resistance, Antibiotics, Dental public health, Analgesics, Anxiolytics, Dental surveys, Dental prescribing

## Abstract

**Background:**

Numerous studies of dental antibiotic prescribing show that overprescribing is a worldwide occurrence. The aim of this study was to assess prescribing practices of general dentists in Australia for antibiotics, analgesics and anxiolytics and to determine the extent to which prescribing is in accordance with current guidelines.

**Methods:**

A structured questionnaire was sent to 1468 dentists in Victoria and Queensland in July–August 2018. The questionnaire covered demographics, clinical conditions where dentists prescribe antibiotics, non-clinical factors which influence prescribing, and medicines for anxiolysis and pain relief. Responses were scored using a system based on the current Australian therapeutic guidelines. Logistic regression was used to determine the relative importance of independent variables on inappropriate prescribing.

**Results:**

Three hundred eighty-two responses were received. Overall, 55% of overprescribing of antibiotics was detected, with a range of 13–88% on a routine or occasional basis depending on the scenario. Between 16 and 27% of respondents inappropriately preferenced analgesics over anti-inflammatories for dental pain; 46% of those who prescribed anxiolytic medicines did so inappropriately, with varying regimens and choices outside the guidelines. Years of practice was the main demographic factor influencing prescribing, with recent graduates (0–5 years) generally scoring better than their colleagues for antibiotic prescribing (*p* < 0.05).

**Conclusions:**

Future interventions could be directed towards the appropriate role and use of antibiotics, shortfalls in knowledge and appropriate choices of medicines for pain relief and anxiolysis. Given that the most overprescribing occurred for localised swellings (88%), this area could be focused on in continuing education as well as ensuring it is addressed in undergraduate teaching. Continuing education on the appropriate use of medicines can be targeted at more experienced dentists as well as patients, especially those who expect antibiotics instead of treatment.

**Trial registration:**

University of Melbourne Human Ethics Sub-Committee; ID: 1750768.1.

**Electronic supplementary material:**

The online version of this article (10.1186/s12903-019-0882-6) contains supplementary material, which is available to authorized users.

## Introduction

Antibiotics are prescribed by dentists both therapeutically and prophylactically for the management of odontogenic infections. It is accepted, however, that active dental treatment is generally the most effective way of treating pain and infection. [1] In Australia, the Therapeutic Guidelines Oral and Dental [1] was established in 2007 to provide clinicians with recommendations regarding appropriate prescribing.

Numerous studies of dental antibiotic prescribing show that overprescribing occurs worldwide, where dentists tend to prescribe for unnecessary indications, often without concurrent dental treatment. [2–4] A cross-sectional study in Wales showed that 70.6% of antibiotics were prescribed without an operative intervention, [2] and a prospective study in Belgium showed high prescription rates for localised infections such as periapical abscesses, with 54.2% of antibiotics prescribed without local treatment. [5] Factors including limited clinical time, fulfilling patients’ expectations, inability to come to a diagnosis and avoiding litigation risk have been documented as non-clinical pressures influencing dental prescribing. [6–8] A survey of US endodontists revealed that almost 37% prescribed antibiotics unnecessarily, mostly due to patient expectations. [6]

Antibiotic resistance is a well-established global public health problem; it is likely that dentists are contributing to by overprescribing and inappropriate prescribing. [9, 10] Several longitudinal studies [11–13] and surveys [4, 5] confirm that dentists prefer moderate to broad spectrum antibiotics over those with a more appropriate narrow spectrum. A retrospective audit of antibiotic resistance in severe odontogenic infections showed that the rate of resistance to penicillins was 10.8%, where these patients subsequently required longer hospital stays and had a higher incidence of non-response to initial surgical therapy. [14] Antibiotic stewardship, defined as “an activity that includes appropriate selection, dosing, route, and duration of antimicrobial therapy”, [15] has been well emphasised in the medical field, but less so in the dental industry. [2] The worldwide financial and health impacts on both the individual and the healthcare system are well established, including resultant difficult-to-manage infections and increased hospital stays. [16]

Medicines for pain relief and anxiolytics are also commonly prescribed by dentists. Opioid misuse and abuse and associated harms are also well-established public health issues, where in Australia, pharmaceutical opioid poisoning has now surpassed that of heroin use. [17] A systematic review showed that opioids for non-medical use are predominately sourced through social networks, using valid prescriptions from family or friends. [18] Increasingly, literature has demonstrated that dentists account for a substantial proportion of opioid prescribing. A cross-sectional analysis of dental prescriptions in the USA showed that opioids accounted for around 20% of dental prescription claims, and more than half the dentists prescribed opioids for longer than the recommended duration of three days. [19] A self-reported survey of dentists and endodontists in Canada showed that the rate of prescription of opioids was high, [20] and a recent cross-sectional study of the opioid prescribing practices by dentists in the USA and England showed high rates of prescribing by US dentists, and a range of opioids were prescribed. [21] Concerningly, longitudinal studies also show that use of opioids and benzodiazepines in Australia is increasing. [22, 23]

Additionally, poor adherence to guidelines is common internationally, with several longitudinal studies have shown that dentists’ prescribing in Australia diverge from current recommendations [11, 12, 22, 23] and a prospective study of dentists in Wales showed only 19% of prescriptions were written in accordance with guidelines. [2] Understanding current prescribing habits and the impact of non-clinical factors on prescribing will help the development of targeted interventions to improve prescribing. The aim of this study was therefore to assess the prescribing practices of general dentists in Australia for all major drug classes and to determine the extent to which prescribing is in accordance with current guidelines and evidence-based practice.

## Methods

A structured questionnaire was mailed to 1468 dentists in Australian states of Victoria and Queensland in July–August 2018, with a stamped return envelope. The total number of practitioners registered with the Australian Health Practitioner Regulation Agency in these states was 7551 in June 2018. [24] As the degree of dental antibiotic overprescribing is currently unknown in Australia, the proportion 0.5 was used as it provides the most conservative estimate of the sample size, and with a degree of accuracy of 0.05 and confidence interval of 95%, a sample size of 367 responses is needed. [25] Allowing for 25% response rate, 1468 (367 × 4) surveys were proportionally distributed, 675 in Queensland and 793 in Victoria. Contact details were obtained from publicly available dental practice websites. Participants were chosen by the location of their dental practice. Ten localities were selected across the states, distributed evenly among the socio-economic index for area (SEIFA) rankings in order to sample dentists working in low, middle and high SEIFA locations. [26] A proportional number of surveys (Victoria: 70; Queensland:84) were sent to dentists practising in a rural location, as classified by the ABS (Victoria: 9%; Queensland: 12%). [27] Ethics approval was obtained from The University of Melbourne Human Ethics Sub-Committee (ID: 1750768.1).

The questionnaire was based on previous surveys [6, 8] with some additional questions about pain relief medicines, anxiolytic prescribing and sources of drug information. The terminology was modified slightly for the Australian context. The first section sought demographic details, including sex, location of training (Australian- or overseas-trained), years of experience since graduation and postcode of work location. The second section investigated clinical conditions where dentists normally prescribe antibiotics for therapeutic reasons, including irreversible pulpitis, pulp necrosis with varying degrees of symptoms, pulp necrosis with acute apical periodontitis and a localised swelling, pulp necrosis with swelling and systemic spread such as cellulitis, the routine use of antibiotics prior to starting root canal treatment, the routine use of antibiotics after starting root canal treatment, alveolar osteitis and the re-implantation of avulsed teeth. Only two clinical conditions (pulp necrosis with acute apical periodontitis and systemic spread, and the re-implantation of avulsed teeth) required antibiotics according to the indications listed in Therapeutic Guidelines. [1] Antibiotics are recommended in the Australian guidelines for avulsion for prophylactic purposes as they can help reduce healing complications such as inflammatory root resorption. [1] Response options were “Yes”, “Occasionally” or “No” to antibiotic prescription.

This section also explored four factors that may influence prescribing: time pressure, local anaesthetic being ineffective due to irreversible pulpitis, patient’s expectations and inability to arrive at a diagnosis. Response options were “Always”, “Occasionally” or “Never” to antibiotic prescription. An “incorrect prescribing score” was calculated by adding incorrect responses from the fifteen antibiotic prescribing questions. Scoring was based on recommendations by the Therapeutic Guidelines and the known pharmacology of the medicines prescribed.

In the third section, dentists were asked to indicate what and how they would normally prescribe for anxiety. They were also asked if they combined medicines for anxiety and if they routinely used nitrous oxide or methoxyflurane. Fourthly, dentists were asked to specify which medicines they normally prescribe for mild and moderate-to-severe pain. The final part of the survey investigated the common sources of therapeutic information used by dentists. The questionnaire is included as an Additional file 1.

A scoring system was developed for each of the 24 questions on medicine use, with most questions having options of correct or incorrect answer. Questionnaires with more than three missing responses were excluded from the analysis.

Data were analysed with IBM SPSS (version 25) software, using descriptive statistics and logistic regression for multivariate analysis. Additionally, to better understand the association between the combination of socio-demographic and work variables and overall incorrect antibiotic prescribing score, a stepwise multiple linear regression analysis was performed. All *p*-values < 0.05 were considered significant.

## Results

Of the 1468 mailed surveys, 403 questionnaires were returned (382 usable responses) satisfying the required sample size (367). Demographic details are shown in Table 1. The almost even proportion of male and female respondents, spread of responses across years of clinical experience, and division by state reflects the demographic parameters recorded by the Australian Health Practitioner Regulation Agency. [24] Responses from a range of SEIFAs, with 10% being from practitioners working in rural areas reflects statistics from the ABS. [26, 27]

**Table 1:** Demographic details

	N*	%
Number of respondents	382	
Gender		
Male	208	54
Female	174	46
Location of training		
Australia	302	79
Overseas	80	21
Years of practising		
0 to 5	70	18
6 to 10	70	18
11 to 20	103	27
21 to 30	64	17
30+	75	20
State		
Victoria	213	56
Queensland	169	44
Work location		
Urban	344	90
Rural	38	10
SES of practice location		
Low - 1, 2, 3	54	14
Middle - 4, 5, 6, 7	152	40
High - 8, 9, 10	176	46

*N: number of responses

.

## Antibiotic prescribing

For clinical and non-clinical uses of antibiotics, participants obtained an overall mean prescribing score of 7.2 (SD 2.8; range: 1–14) incorrect answers, with 23% of dentists obtaining 10 or more, which included both unnecessary use and underuse. For overprescribing only, dentists obtained a mean of seven incorrect responses from 13 questions, i.e. 55% antibiotic overprescribing.

### Clinical uses of antibiotics

Responses for the therapeutic and non-clinical uses of antibiotics are detailed in Table 2, which shows the overall percentage of over- or under- prescribing by respondents on a routine or occasional basis (13–88% depending on the clinical scenario). Significant unnecessary use of antibiotics was evident, the most overprescribing (88%) was seen for acute apical periodontitis with localised swelling (Question 6), followed by use of antibiotics for pulp necrosis with acute apical periodontitis reducing a localised swelling prior to starting root canal treatment (Question 8), where 77% of respondents would habitually or occasionally use antibiotics prior to commencing treatment. Overall, 27–50% of dentists reported that they routinely or occasionally prescribe antibiotics for irreversible pulpitis (Questions 1 and 2) and 52% reported routine or occasional prescribing for pulp necrosis with acute apical periodontitis in addition to local measures (Question 4). Although alveolar osteitis is a failure of healing due to lysis of the blood clot after an extraction, [1] 40% of participants would always or sometimes prescribe antibiotics for management (Question 10). The vast majority (96%) would always prescribe appropriately for odontogenic infections that have systemic spread such as cellulitis (Question 7) although only 75% would always prescribe for re-implantation (Question 11).

**Table 2:** Descriptive analysis showing the percentage of dentists who would prescribe antibiotics for the following clinical and non-clinical scenarios

	Yes	Occasionally	No
Q1. IP, moderate/severe symptoms	7	20	73
Q2. IP/AAP, moderate/severe symptoms	15	35	50
Q3. PN/CAP, no swelling, no/mild symptoms	2	11	87
Q4. PN/AAP, no swelling, moderate/severe symptoms	15	37	48
Q5. PN/CAP, sinus tract present, no/mild symptoms	9	21	70
Q6. PN/AAP, localised swelling present, moderate/severe symptoms	50	38	12
Q7. PN/AAP, swelling present, systemic spread present (eg cellulitis)^a^	96	1	3
Q8. PN/AAP, swelling present, no systemic spread, use antibiotics prior to starting RCT to reduce the swelling	38	39	23
Q9. PN/AAP, to prescribe antibiotics routinely after RCT	2	14	84
Q10. Alveolar osteitis (dry socket)	13	27	60
Q11. Re-implantation of avulsed teeth^a^	75	14	11
	Always	Occasionally	Never
Q12. Time pressure	8	69	23
Q13. IR, delaying treatment due to ineffective local anaesthetic	24	54	22
Q14. Patient’s request for antibiotics instead of treatment	2	80	18
Q15. Inability to come to a definitive diagnosis	5	62	33

^a^Antibiotics are indicated according to the Australian Therapeutic Guidelines

*IP* Irreversible pulpitis, *AAP* Acute apical periodontitis, *PN* Pulp necrosis, *CAP* Chronic apical periodontitis, *RCT* Root canal treatment

### Non-clinical influences

Overprescribing due to other factors e.g. time pressure, local anaesthetic being ineffective and inability to diagnose played a significant role, being reasons for prescribing for 67–78% of dentists on a regular or occasional basis. (Table 2). Patients’ requesting antibiotics instead of treatment were encountered by 82% of dentists on a routine or occasional basis.

### Influence of demographic factors

Logistic regression showed that experience was the predominant variable in prescribing, with less experienced dentists (0–5 years) showing a greater likelihood of appropriate prescribing compared to their more experienced colleagues (over 30 years), as shown in Table 3. As all antibiotic questions (Questions 1–15) were subject to logistic regression, only the significant findings were presented in the results to avoid confusion. This was the case for the clinical scenario of pulp necrosis with acute apical periodontitis (Question 4, *p* = 0.005, OR:2.79, 95% CI: 1.36–5.74) and alveolar osteitis (Question 10, *p* = 0.001, OR:4.82, 95% CI: 1.96–11.86). For the routine use of antibiotics after starting root canal treatment, less experienced dentists of 0–5 years (Question 9, *p* = 0.002, OR:7.77, 95% CI: 2.11–28.61) and 6–10 years (*p* = 0.044, OR:2.53, 95% CI: 1.02–6.25) provided more appropriate responses than their colleagues with over 30 years’ experience. The variance explained by the full model for differences on each of the prescribing items ranged from 7 to 13%.

**Table 3:** Logistic regression to identify independent demographic factors that affect appropriate antibiotic prescribing

Question	Demographic factor	Subgroup	P	OR	CI (95%)	Variance
Q4. PN/AAP/no swelling/mild-moderate symptoms	Years of practising	0 to 5	0.005	2.79	1.36–5.74	8.6
Q9. PN/AAP, prescribe antibiotics routinely after RCT	Years of practising	0 to 5	0.002	7.77	2.11–28.61	9.1
		6 to 10	0.044	2.53	1.02–6.25	
Q10. Alveolar osteitis (dry socket)	Years of practising	0 to 5	0.001	4.82	1.96–11.86	13.3
Q11. Re-implantation of avulsed teeth	Years of practising	0 to 5	0.002	0.23	0.09–0.58	6.9
		6 to 10	0.033	0.42	0.19–0.93	
Q14. Patient’s request for antibiotics	Years of practising	6 to 10	0.007	0.23	0.08–0.67	9.7
		21 to 30	0.011	0.29	0.11–0.75	
Q15. Inability to come to a definitive diagnosis	Years of practising	11 to 20	0.02	0.44	0.22–0.88	9
		21 to 30	0.039	0.45	0.21–0.96	
	Victoria/Queensland	Victoria	0.017	0.58	0.37–0.90	
	Urban/Rural	Rural	0.012	2.59	1.24–5.42	

*PN* Pulp necrosis, *AAP* Acute apical periodontitis, *RCT* Root canal treatment

Multivariate analysis confirmed that only years of professional experience had an effect on the overall antibiotic prescribing score (*p* < 0.01), with dentists with 0–5 years of experience having a mean of 6.1 compared to all other years of experience groups, which all scored over 7.4.

## Anxiolytic prescribing

Most dentists (90%) made appropriate choices of anxiolytics (diazepam or temazepam) as indicated by the guidelines, or did not prescribe at all. (Table 4). A small percentage (5%) would refer patients to their medical doctor, and 5% made choices outside the recommended guidelines, including oxazepam, triazolam, alprazolam, midazolam and chloral hydrate. Of the dentists who prescribed anxiolytics or indicated what they would request the patient’s medical doctor to prescribe (*n* = 259), only 54% adhered to the recommended regimen, of a single dose of anxiolytic agent an hour prior to treatment. The regimen used by 34% of respondents who prescribed anxiolytics was to use the anxiolytic the night prior and an hour before the appointment. The remaining 12% gave other varied regimens (which included regimens where the drug was used for more than two doses and up to three days prior to the appointment), or chose an anxiolytic outside the guidelines. Only 1% of respondents would combine medicines for anxiolysis. Nitrous oxide and methoxyflurane were routinely used by 34 and 9% of dentists respectively.

**Table 4:** Anxiolytic and pain relief prescribing choices

	N*	%
Percentage of dentists responses when asked about their choices of medicines for anxiolysis		
Does not prescribe	93	24
Diazepam and/or temazepam	252	66
Refer to medical doctor	19	5
Other	18	5
Appropriateness of dose and/or regimen of those who prescribed anxiolytics		
Appropriate prescription of anxiolytic (single dose one hour prior to the procedure)	141	54
Two doses of anxiolytic used (night before and prior to the procedure)	87	34
More than 2 doses used, or choice of anxiolytic outside the guidelines	31	12
Percentage of dentists who combined > 1 anxiolytic		
Yes (combined > 1)	4	1
No (did not combine/did not prescribe)	378	99
Percentage of dentists who use nitrous oxide		
Uses nitrous oxide	131	34
Does not use nitrous oxide	251	66
Percentage of dentists who use methoxyflurane		
Uses methoxyflurane	34	9
Does not use methoxyflurane	348	91
Percentage of dentists responses when asked about their choices of medicines for pain relief		
Choices for a simple extraction (mild pain relief)		
Prescribes an NSAID in addition to analgesics	299	78
Prescribes analgesics only	61	16
Inappropriate choice of analgesia or analgesia not recommended in TG	15	4
No prescription	7	2
Choices for multiple extractions (moderate-severe pain relief)		
Prescribes an NSAID in addition to analgesics	239	63
Prescribes analgesics only	105	27
Inappropriate choice of analgesia or analgesia not recommended in TG	36	9
No prescription	2	1

*N: number of responses

## Prescribing medicines for pain relief

The majority of dentists (63–78%) made appropriate choices of analgesia for both mild and severe pain relief, choosing an appropriate anti-inflammatory (ibuprofen, aspirin or naproxen) in addition to other analgesics. Ibuprofen and aspirin are recommended in the guidelines, and naproxen is also a reasonable choice considering its known pharmacology and acceptable adverse effect profile. A significant number (16–27%) would prescribe analgesics only without anti-inflammatories for mild and severe pain relief respectively. Only 4–9% of dentists would routinely prescribe inappropriate analgesics, including diclofenac, tramadol, mefenamic acid, ketoprofen, codeine, oxycodone, dexamethasone and diazepam. (Table 4).

## Sources of information

Of the dentists who responded regarding sources of therapeutic information (*n* = 272), the majority preference the recommended Therapeutic Guidelines [1] (69%); 28.8% of respondents ticked more than one option (which was not allowed by the question), so these answers were excluded. As 110 responses had to be excluded, these results were omitted.

## Discussion

This is the first study in Australia to assess the various therapeutic uses of antibiotics, anxiolytics and medicines for pain relief by dentists since the establishment of national guidelines and to determine the influence of various non-clinical factors on antibiotic prescribing.

The study showed a gross overuse of antibiotics for both therapeutic and non-therapeutic reasons. The majority of dentists prescribed appropriately for anxiolysis, although a small but significant number made choices outside the recommended guidelines or employed an inappropriate regimen. Similarly, for pain relief, most dentists would prescribe appropriately but a substantial number inappropriately preferenced the use of other analgesics over anti-inflammatories, and others would prescribe strong opioids not recommended in the guidelines.

A significant degree of unnecessary use of antibiotics for inappropriate therapeutic indications was evident, similar to other findings worldwide. [2–5] It has been shown that dentists tend to prescribe for indications where antibiotics are not required, including alveolar osteitis, [1] irreversible pulpitis [28] and varying stages of pulpal pathology where the infection is localised. [29, 30] Systematic reviews and other studies have documented the need for antibiotics with dental treatment only when there is evidence of systemic spread or a spreading superficial infection, [29, 30] and the most effective management of localised infections is with active treatment only. Given that the most overprescribing occurred for localised swellings (88%), this could be clarified in continuing education and undergraduate teaching. The misconception that antibiotics can be given to help reduce a localised swelling to make the local anaesthetic more effective should be rectified, as it is established that treatment of an acute odontogenic infection with antibiotics alone can be deleterious because of the risk of worsening infection with development of airway compromise. [1]

The amount of experience of the dentist was a significant factor in overprescribing, with recent graduates prescribing the most appropriately, probably according to their recent teaching. Other factors including postgraduate education and the type of practice (solo or group) have been shown in other similar surveys [6, 31] to produce a positive association with appropriate prescribing, were not asked in this survey. A recent qualitative study on perceptions and reasons for prescribing antibiotics for therapeutic uses revealed that for conditions such as irreversible pulpitis, localised odontogenic infections and alveolar osteitis where antibiotics are not warranted, dentists tended to prescribe antibiotics based on the severity of the patient’s symptoms, rather than clinical signs. [32] The study also revealed that there was a strong desire by dentists to give distressed patients who were in pain the impression that the dentist was doing everything possible to resolve their symptoms so patients would consequently feel that they were well managed, and the prescribing of antibiotics was one such method. [32]

This present study also revealed that other pressures, including time pressure and the inability to arrive at a diagnosis influenced prescribing for the majority of respondents. These findings are broadly supported by other surveys, where a prospective study in Wales showed that the odds of a dentist prescribing antibiotics when there was limited clinic time was ten-fold, [2] and 39% of dentists in Switzerland would occasionally prescribe antibiotics when they were uncertain of a diagnosis. [7] Time pressure is difficult to rectify as it is not practical to allow extended time for unexpected patients while maintaining a sustainable dental practice. Many other non-clinical factors have emerged in the literature, including medico-legal considerations, [5, 33] fear of online criticism, and pressure from assistant staff to prescribe. [32] With the increasing public health threat of antibiotic-resistant bacteria, all prescribers have a professional responsibility for restraint in antibiotic use. [9]

While the vast majority of dentists made appropriate choices of anxiolytic medicines, there was a small but significant number who would use other anxiolytics which are not recommended in the guidelines, with the other choices being lorazepam, alprazolam, oxazepam, midazolam and chloral hydrate. The high potency benzodiazepine, alprazolam, registered for use in Australia for anxiety and panic disorders, [34] has unique pharmacokinetic properties including a short half-life and rapid absorption lending it to increased withdrawal symptoms including significant rebound anxiety. [35, 36] Furthermore, alprazolam particularly causes increased levels of dopamine in the central nervous system (CNS), similar to stimulants and other drugs which have abuse potential. [36] It should therefore be discouraged for use in dental practice given its high misuse liability, [36] classifying it is a controlled drug. [34]

A small but significant percentage of dentists indicated they would prescribe benzodiazepines for several doses, with some up to three days prior to the procedure and some prescribing increased dose quantities. A discussion paper from the Dental Board of Australia states that “minimal sedation (anxiolysis) is the use of a single low dose oral sedative drug,” as advised by the International Federation of Dental Anaesthesiology. [37]

While the majority of dentists indicated they would prescribe medicines for pain relief appropriately, preferencing the use of non-steroidal anti-inflammatory drugs (NSAIDs), 16–27% would prescribe an analgesic only. NSAIDs are the preferred choice and most effective for dental pain as they inhibit the inflammatory response. [38, 39] A qualitative study has also shown that NSAIDs are superior to paracetamol for pain relief after dental surgery, [40] and a double-blind randomised controlled trial showed that analgesic doses of codeine had no effect on pain scores after surgical third molar extractions, compared to ibuprofen and paracetamol. [41] In addition, given the established misuse of pharmaceutical opioids in Australia [42] and other countries, and that leftover dental opioid prescriptions can be a source of diversion, [18] dentists should only prescribe opioids if anti-inflammatories and paracetamol have not been effective and should ensure a true therapeutic need exists. In comparison to English dentists who only prescribe the codeine derivative dihydrocodeine, [21] Australian dentists prescribe a range of opioids, despite having guidelines in place. This was also reflected in previous studies of Australian dental prescribing. [22, 23] One measure to assist with the monitoring of drugs prone to misuse is the Safe Script program, [43] which will be mandatory in the Australian states of Victoria and Tasmania from April 2020. This initiative allows prescribers to access information on all prescriptions of drugs prone to misuse that are dispensed for each patient to help assist with “doctor shopping” and to prevent the increased acquisition of these drugs from multiple prescribers. Dentists are currently not included on the program and the initiative is only implemented in two Australian states. Including dentists may help prevent opioid or benzodiazepine prescribing to people who are seeking these drugs for non-medical use.

A range of medicines were listed by respondents as routine recommendations for dental pain, including diclofenac, tramadol, mefenamic acid, ketoprofen, codeine, oxycodone, dexamethasone and diazepam, the latter having no indication for pain relief. [34] Previous longitudinal studies of dental prescribing confirm a significant number of dispensed prescriptions for diclofenac, [22, 23] which is not recommended as diclofenac carries the highest risk of cardiovascular adverse effects of all nonselective NSAIDs, [44] even with short-term use (1–7 days). [22, 45] A qualitative survey on dental prescribing choices revealed that tramadol was often recommended for patients who could not tolerate codeine. [32] It should be noted that similar to codeine, tramadol is a pro-drug and requires biotransformation by cytochrome P450 2D6. [34, 39] Patients who have inherited two non-functional alleles of P450 2D6 will therefore have poor analgesia from both opioids. [39]

The study has some limitations. Dentists may have provided professionally acceptable responses, introducing bias. Variance in the multivariate analysis was low (7–13%), so there are likely to be other variables that affect prescribing which were not addressed, such as the collaborative effect of dentists as determined from previous studies. [32] Nonetheless, the strength of the study is the sufficient sample size, with varied demographic characteristics, which likely made this sample reflective of the population.

## Conclusion

Future interventions could be directed towards education about the appropriate use of antibiotics and appropriate drug choices and regimens for pain relief and anxiolysis. Increased focus could also be targeted to patient education, as is being addressed by National Prescribing Service Medicinewise. [46] Drug knowledge of the toxicity, adverse effects, appropriate patient selection and drug interactions could also be made more readily available for dentists to make informed and safe decisions when considering the prescription of drugs not indicated in the guidelines.

## Additional file

Additional file 1: Therapeutics Survey. (DOCX 19 kb)

## Data Availability

The survey and datasets generated from this study are available from the corresponding author on reasonable request.

## Abbreviations

NSAIDs: Non-steroidal anti-inflammatory drugs
