# Supplementary material for: A survey of prescribing practices by general dentists in Australia
Source: BMC Oral Health. 2019 Aug 22;19:193. doi: 10.1186/s12903-019-0882-6 (PMC6704722; doi:10.1186/s12903-019-0882-6)
Supplement: Supplementary file 1 — Therapeutics Survey. (DOCX 19 kb) [file 12903_2019_882_MOESM1_ESM.docx]

**THERAPEUTICS SURVEY**

**Demographic Questions**

□ Male □ Female

Where did you complete your dental training?

□ Australia □ Overseas

How many years have you been working?

□ 0-5 □ 6-10 □ 11-20 □ 21-30 □ 30+

Postcode of your primary work location:………………………………..

**THERAPEUTIC QUESTIONS**

**Infections – Antibiotic prescribing**

Please indicate (tick yes or no) if you would prescribe antibiotics **therapeutically** for the following clinical indications, in addition to regular dental treatment (unless otherwise stated).

| CLINICAL INDICATIONS FOR THERAPEUTIC USE (TO TREAT INFECTION) | YES | OCCASIONALLY | NO |
| --- | --- | --- | --- |
| 1. Irreversible pulpitis, moderate/severe symptoms |  |  |  |
| 2. Irreversible pulpitis with acute apical periodontitis, moderate/severe symptoms |  |  |  |
| 1. Pulp necrosis with chronic apical periodontitis, no swelling, no/mild symptoms |  |  |  |
| 1. Pulp necrosis with acute apical periodontitis, no swelling, moderate/severe symptoms |  |  |  |
| 1. Pulp necrosis with chronic apical periodontitis, sinus tract present, no/mild symptoms |  |  |  |
| 1. Pulp necrosis with acute apical periodontitis, localised swelling present, moderate/severe symptoms |  |  |  |
| 1. Pulp necrosis with acute apical periodontitis, swelling present, systemic spread present (eg cellulitis) |  |  |  |
| 1. Pulp necrosis with acute apical periodontitis, swelling present, no systemic spread but to use antibiotics prior to starting root canal treatment/extraction to reduce the swelling and to make the local anaesthetic more effective |  |  |  |
| 1. Pulp necrosis with acute apical periodontitis, to prescribe antibiotics routinely after starting root canal treatment |  |  |  |
| 1. Alveolar osteitis (dry socket) |  |  |  |
| 1. Re-implantation of avulsed teeth |  |  |  |

To further investigate the common pressures experienced by general dentists, please indicate if you would prescribe antibiotics in these clinical scenarios (please tick yes, occasionally or no)

| CLINICAL SCENARIO | ALWAYS | OCCASIONALLY | NEVER |
| --- | --- | --- | --- |
| 1. If you are pressed for time and the patient has a localised odontogenic infection, you would prescribe antibiotics and make another appointment for the patient |  |  |  |
| 1. If the local anaesthetic is ineffective and/or the patient has severe symptoms of irreversible pulpitis, you would prescribe antibiotics and delay treatment to another time |  |  |  |
| 1. Do your patients request for antibiotics instead of treatment? |  |  |  |
| 1. If you are unable to come to a definitive diagnosis and the patient has symptoms of a likely odontogenic infection (eg pain at night, pain on percussion) |  |  |  |

16. Are you aware of the problems associated with antibiotic resistance?

□ Yes □ No

17. If yes, does the knowledge of the problems associated with antibiotic resistance affect your prescribing practices?

□ Yes □ No

**Anxiolysis - Anxiolytic prescribing**

18. What medication(s) would you normally prescribe for anxiolysis? Please tick which apply.

□ Diazepam □ Temazepam □ Lorazepam □ Oxazepam

□ Zolpidem (Stilnox) □ Other (please list)...........................................

19. Please indicate what dose and regimen for each medication (for an adult with no other medical conditions/medications): *For example: Diazepam 5mg the night before the procedure, followed by 10mg one hour before the procedure.* ............................................................................................................................................................................................................................................................................................................................................

20. If you prescribe the above medication(s), do you find that prescribing more than one drug for concurrent use works more effectively for your patients in clinical practice? If yes, please indicate your preferred dosing regimen (for an adult with no other medical conditions/medications). *For example: Diazepam 5mg the night before the procedure, followed by Oxazepam 30mg, one hour prior to the procedure.*

............................................................................................................................................................................................................................................................................................................................................

21. Do you use (tick which apply): □ nitrous oxide □ methoxyflurane

**Pain relief – Analgesic and Anti-inflammatory prescribing**

What medications do you prescribe for pain relief, for the following two scenarios?

22. For a **simple extraction** (where mild pain relief is required)? Please tick which apply.

□ Panadeine forte (paracetamol 500mg with codeine 30mg)

□ Panadeine (paracetamol 500mg with codeine 8mg)

□ Mersyndol (paracetamol 450mg/codeine 9.75mg/doxylamine 4.5mg)

□ Paracetamol □ Oxycodone □ Tramadol □ Nurofen(ibuprofen)

□ Aspirin □ Naproxen □ Indomethacin □ Voltaren(diclofenac) □ Ketoprofen □ Piroxicam □ Other (please list)…………………………

23. For **multiple extractions/surgical extraction** (where moderate-severe pain relief is required)? Please tick which apply.

□ Panadeine forte (paracetamol 500mg with codeine 30mg)

□ Panadeine extra (paracetamol 500mg with codeine 8mg)

□ Mersyndol (paracetamol 450mg/codeine 9.75mg/doxylamine 4.5mg)

□ Paracetamol □ Oxycodone □ Tramadol □ Nurofen(ibuprofen)

□ Aspirin □ Naproxen □ Indomethacin □ Voltaren(diclofenac)  □ Ketoprofen □ Piroxicam □ Other (please list)…………………………

**SOURCES OF INFORMATION ON THERAPEUTICS**

24. What is your main source of information on therapeutics and prescribing? (tick one box only, the most common source for you)

□ Colleagues

□ What has worked in the past for your patients

□ Therapeutic guidelines Oral and Dental

□ Other drug references such as MIMS

25. Which sentence best describes what you think of the pharmaceutical benefits scheme (PBS) for dentists: (tick one)

□ I am allowed to prescribe any medication on that list since it is the PBS for dental prescribing

□ I see it as a guide only, and I also prescribe medicines that are not on that list (private prescriptions) if I think that is best for the patient

□ I prescribe what I think is best without considering the PBS

**THANK YOU FOR YOUR TIME**
